# Supplementary material for: Changes in the amount of nutrient of packaged foods and beverages after the initial implementation of the Chilean Law of Food Labelling and Advertising: A nonexperimental prospective study
Source: PLoS Med. 2020 Jul 28;17(7):e1003220. doi: 10.1371/journal.pmed.1003220 (PMC7386631; doi:10.1371/journal.pmed.1003220)
Supplement: S1 Table — (DOCX) [file pmed.1003220.s003.docx]

|  | 1st phase (2016) | 2nd phase (2018) | 3rd phase (2019) |
| --- | --- | --- | --- |
| Per 100g of solids |  |  |  |
| Energy [kcal] | 350 | 300 | 275 |
| Total sugars [g] | 22.5 | 15 | 10 |
| Saturated fats [g] | 6 | 5 | 4 |
| Sodium [mg] | 800 | 500 | 400 |
| Per 100mL of liquids |  |  |  |
| Energy [kcal] | 100 | 80 | 70 |
| Total sugars [g] | 6 | 5 | 5 |
| Saturated fats [g] | 3 | 3 | 3 |
| Sodium [mg] | 100 | 100 | 100 |
