# Supplementary material for: Changes in the amount of nutrient of packaged foods and beverages after the initial implementation of the Chilean Law of Food Labelling and Advertising: A nonexperimental prospective study
Source: PLoS Med. 2020 Jul 28;17(7):e1003220. doi: 10.1371/journal.pmed.1003220 (PMC7386631; doi:10.1371/journal.pmed.1003220)
Supplement: S2 Table — T0: preimplementation period, January to February 2015 + January to February 2016 (cross-sectional n = 4,055, longitudinal n = 1,915). T1: postimplementation period, January to February 2017 (cross-sectional n = 3,025, longitudinal n = 1,915). (DOCX) [file pmed.1003220.s004.docx]

| Food or beverage group | Specific foods or beverages included |
| --- | --- |
| Beverages  T0, n = 686  T1, n = 482  Longitudinal, n = 326 | 100% fruit juices, almond milk with or without sugar, aromatized/flavored waters with or without sugar, carbonated water with or without sugar, soft drinks with or without sugar, frozen and not frozen fruit pulp, fruit-based juices (nectar and others) with or without sugar, fruit juices with sugar, herb tea or “yerba mate” with sugar or without sugar, instant coffee or coffee drinks with or without sugar (in a pot or bag), isotonic beverages (liquid and powder) with or without sugar, liquid tea with or without sugar, non-carbonated water, powder/concentrated juices with or without sugar, soy milk with or without sugar, other beverages (all types). |
| Milks and milk-based drinks  T0, n = 201  T1, n = 103  Longitudinal, n = 76 | Liquid flavored semi-skimmed milk, liquid flavored skimmed milk, liquid semi-skimmed milk, liquid skimmed milk, liquid whole milk, milk drinks (i.e., beverages with at least 30% of their volume being milk, according to the Chilean food regulation), powder semi-skimmed milk, powder skimmed milk, powder whole milk, toddler milk (for 1-5 y children), other liquid dairy products. |
| Yogurts  T0, n = 312  T1, n = 272  Longitudinal, n = 181 | Light or diet flavored yogurt, light or diet yogurt with fruits and/or nuts, light or diet yogurt with cereals, yogurt with fruits and/or nuts, yogurt with sweet sauces, yogurt with cereals, flavored yogurt, plain yogurt. |
| Breakfast cereals  T0, n = 148  T1, n = 125  Longitudinal, n = 67 | Baked corn cereals, chocolate cereals, chocolate cereal bars, dry fruits cereal bars, fiber-enriched cereals, flakes/balls cereals, fruit cereal bars, granola, muesli, oat meal, regular cereal bars, other cereals. |
| Sweet baked products  T0, n = 198  T1, n = 173  Longitudinal, n = 118 | Alfajores, big cookies, biscuits with filling, brownies, cakes, chilean pastries, Christmas bread, cuchuflies, filled wafers, frozen biscochos, muffins, sweet biscuits, wafers without filling, other biscochos and rolls. |
| Desserts and ice creams  T0, n = 437  T1, n = 333  Longitudinal, n = 230 | Canned fruits with or without sugar, cold desserts, diet ice cream, flan, frozen desserts, fruit and vegetable chips, fruit compote, ice cream popsicles, ice cream, leche asada, milk rice pudding, water-based ice creams, water-based ice cream popsicles, yogurt ice cream, other ice creams. |
| Candies and sweet confectioneries  T0, n = 391  T1, n = 445  Longitudinal, n = 216 | Bonbons, bubble gum filled candies, bubble gums, candies, candy pops, chewy candy, chocolate covered dried fruits, chocolates, dried fruits with caramel coating, gummies, jellies, liquid filled bubble gums, manjar bars, manjar pop, marshmallows, sweet popcorn, toffees, other candies and sweet confectionery. |
| Sweet spreads  T0, n = 165  T1, n = 115  Longitudinal, n= 73 | Caramel, chantilly cream and other kind of creams used for pastry as coconut cream or fresh cream, chocolate chips, chocolate sparks (for pastry), chuchoca, condensed milk, evaporated milk, flavor essences for pastry (lemon, vanilla, etc.), flavor essences, flavored powders for milk, flavored syrups, frostings, grated coconut (for pastry), honey (all kinds), jam (all kinds), manjar, other sweet spread, sauces, or products used for pastry. |
| Savory baked products  T0, n = 100  T1, n = 81  Longitudinal, n = 61 | Dough for sopaipillas or others, frozen bread/dough, light or diet packaged white bread loaf, packaged white bread loaf (all kinds), packaged whole wheat bread loaf (all kinds), savory cookies, soda crackers, sopaipillas, tortillas (wheat and corn tortillas), other savory baked products. |
| Savory snacks  T0, n = 69  T1, n = 70  Longitudinal, n = 29 | Potato chips, ramitas, salty soufflitos, other snacks. |
| Savory spreads  T0, n = 210  T1, n = 174  Longitudinal, n = 112 | Butter (regular and light), cesar dressing, chicken/meat/seafood broth (not vegetable broth), chili pepper, ketchup, margarine (regular and light), mayonnaise, mustard, peanut butter, tomato sauce, vegetable broth, other dressings, other spreads. |
| Cheeses  T0, n = 109  T1, n = 117  Longitudinal, n = 60 | Blue cheese, buttery cheese, cream and spreadable cheeses, fresh cheeses, grated cheese, light cheeses, semi-hard cheese, other cheeses. |
| Ready-to-eat meals  T0, n = 243  T1, n = 223  Longitudinal, n = 109 | Canned vegetables, canned legumes, empanadas, frozen meals, humitas, instant mashed potatoes, meat substitutes, pasta-based preparations that can be easily cooked (i.e., macaroni and cheese), pizzas, potato gnocchi (all kinds), ready to serve legumes, ready to serve vegetables, rice-based preparations that can be easily cooked (i.e., chaufa rice). |
| Sausages  T0, n = 362  T1, n = 142  Longitudinal, n =120 | Bacon, chorizo, chorizillo, ham (all kinds), longaniza, salami, other sausages. |
| Nonsausage meat products  T0, n = 297  T1, n = 101  Longitudinal, n = 77 | Breaded meat, fresh or frozen (chicken, pork, beef, turkey), breaded fish, fresh or frozen, canned seafood and fish, fish hamburgers, fish nuggets, marinated fish, fresh or frozen, marinated meat, fresh or frozen (chicken, pork, beef, turkey), hamburgers (chicken, pork, beef, turkey), nuggets (chicken, pork, beef, turkey), other processed meats (chicken, pork, beef, turkey, fish). |
| Soups  T0, n = 125  T1, n = 69  Longitudinal, n = 57 | Instant soups, legume-cream soups, powdered soups (to prepare). |
