# Supplementary material for: Changes in the amount of nutrient of packaged foods and beverages after the initial implementation of the Chilean Law of Food Labelling and Advertising: A nonexperimental prospective study
Source: PLoS Med. 2020 Jul 28;17(7):e1003220. doi: 10.1371/journal.pmed.1003220 (PMC7386631; doi:10.1371/journal.pmed.1003220)
Supplement: S3 Table — (DOCX) [file pmed.1003220.s005.docx]

| Values of energy and nutrients of concern considered outliers (definition based on the visual inspection of the density curves by specific food group) were omitted (154 values omitted).  The following criteria were used (values are per 100 g, except for beverages, milks and milk-based drinks, and soups which values are per 100 mL):   1. *Energy* amount > 200 kcal for beverages, and milks and milk-based drinks; > 600 kcal for desserts and ice creams; > 900kcal savory baked products, and sausages; > 500kcal for cheeses (n = 33). 2. *Sugars* amount >20 g for savory snacks; > 30 g for milks and milk-based drinks; > 30 g for beverages; > 40 g for savory baked products; > 50 g for desserts and ice creams; > 88.5 g for sweet spreads; > 30 g for savory spreads; > 26 g for ready-to-eat meals (n = 38). 3. *Saturated fats* amount > 20 g for savory baked products; > 25 g for sweet baked products; > 9 g for yogurts, and milks and milk-based drinks; > 60 g for ready-to-eat foods; and > 45 g for sausages (n = 8). 4. *Sodium* amount > 150 mg for yogurts, and milks and milk-based drinks; > 250 mg for dessert and ice creams; > 320 mg for beverages; > 600 mg for sweet baked products; > 850 mg for breakfast cereals; > 1000 mg for candy and sweet confectionery, and for sweet spreads; > 1200mg for savory baked products; > 8000 mg for savory spreads (n = 75). |
| --- |
