# Supplementary material for: Changes in the amount of nutrient of packaged foods and beverages after the initial implementation of the Chilean Law of Food Labelling and Advertising: A nonexperimental prospective study
Source: PLoS Med. 2020 Jul 28;17(7):e1003220. doi: 10.1371/journal.pmed.1003220 (PMC7386631; doi:10.1371/journal.pmed.1003220)
Supplement: S1 Text — (DOCX) [file pmed.1003220.s006.docx]

**Chilean INTA-UNC collaborative research**

**Name:** Marcela Reyes

**Date:** February 2019

**Title of Proposed Project:** Changes in content of critical nutrients on packaged foods after a short-term implementation of the Chilean Food Labelling and Marketing Law

**Project Objective (1-3 sentences):** Among packaged foods available in supermarkets from Santiago after the implementation of the Food Labelling and Marketing Law to describe changes in nutritional quality (ie. content of energy, sugars, fats, sodium)), and in the proportion of foods considered regulated (1^st^ phase), stratified by big food categories.

**Research Question(s) and Hypotheses:** The implementation of the regulation (first phase) induced reformulation of packaged foods aimed skipping the regulated status; such reformulation was not homogeneous among different food categories.

**Rational/Background (brief description, including initial review of published literature, 300 words or less):** The Chilean government implemented in June 2016 the Food Labelling and Marketing Law which implies that foods with excessive amounts of energy, sugars, saturated fats and sodium must use a warning label stating such condition in the case of packaged foods and cannot be sold or offer into schools or use marketing strategies targeted to children under 14y. In order to avoid being in the regulated category, food items may have reformulated, decreasing the content of energy and regulated nutrients. The extent of reformulation may have varied according to the anticipated ‘regulated’ condition at baseline (ie. food items above the anticipated cutoff, prior to the implementation, are more likely to reformulated than the ones that will not be regulated).

**Data Sources (be specific):** Nutrient fact panels information derived from photos taken in the supermarket in February 2015 or 2016 (baseline), and February 2017 (linked in order to get a longitudinal data set).

**Key Independent and Dependent Variables:**

Dependent variables:

-Content in 100 g or mL of energy [kcal], sugars [g], sodium [mg] and saturated fats [g] pre & post

-Proportion of regulated foods for the 1^st^ phase pre & post

-Delta (pre-post) in energy [kcal], sugars [g], sodium [mg] and saturated fats [g] distributions (or the % of change from baseline distribution).

Independent variables:

-Year of data collection: 2015-2016 (pre) vs 2017 (post)

-Being above the cutoffs for the 1^st^ phase at baseline (2015-2016)

Other variables:

-Food groups

**Brief overview of Methods and Analyses (300 words or less):** We will work with two type of data set: (i) the overall samples at baseline and in 2017, and (ii) a longitudinal sample, including only food with pre and post data. In the first data set we will compare (independent and paired bivariate analyses) the p50 (p25-27) of energy [kcal], sugars [g], sodium [mg] and saturated fats [g], and the proportion of regulated foods for the 1^st^ phases (by food category). In the second data set, we will confirm the results derived from first data set and will model the association between the anticipated condition of being high in <nutrient> (yes/no) and changes (delta or % of change) of <nutrient>, according to food groups (after testing for interaction).

**Policy Implications** (1-3 sentences on why would other scientists in your area care? Why would the public health community at large care? Policymakers?): FoP and other regulatory measures are expected to have effect at different levels, one of those being the nutrient quality of available foods. A great global interest has been put in learning whether in the Chilean experience there was reformulation, in which extend and in which products.

**ADDITIONAL INFORMATION**

**Names of co-authors:** Rebecca Kanter, Stephanie Vandevijvere, Barry Popkin, Lindsey Smith-Taillie, Camila Corvalan

**Is presentation at a professional meeting anticipated:** **Yes  No X**

**If yes, the meeting title is:**

**Abstract Due Date:**

## Name of target journals for submission (top 3): Lancet, Plos, BMJ, Nutrients

**Timetable for completion of first draft:** June 2019

Table 1. Products included in each food category

|  | Pre-implementation  cross-sectional sample | | Post-implementation  cross-sectional sample | | Longitudinal sub-sample | |
| --- | --- | --- | --- | --- | --- | --- |
|  | N | Liquids [%] | N | Liquids [%] | N | Liquids [%] |
| Total |  |  |  |  |  |  |
| Beverages |  |  |  |  |  |  |
| Sweet baked products |  |  |  |  |  |  |
| Ice cream & desserts |  |  |  |  |  |  |
| Ready to eat meals |  |  |  |  |  |  |
| Solid savory spreads & dressings |  |  |  |  |  |  |
| Liquids savory spreads & dressings |  |  |  |  |  |  |
| Breakfast cereals |  |  |  |  |  |  |
| Candies & sugar confectionary |  |  |  |  |  |  |
| Yogurts, milks & milk-based drinks |  |  |  |  |  |  |
| Sweet spreads |  |  |  |  |  |  |
| Savory baked product |  |  |  |  |  |  |
| Cheese |  |  |  |  |  |  |
| Dried fruits & snacks |  |  |  |  |  |  |
| Non-sausages meats |  |  |  |  |  |  |
| Baby foods |  |  |  |  |  |  |
| Soups |  |  |  |  |  |  |
| Sausages |  |  |  |  |  |  |

In each category the liquid condition was defined according to the unit of measure declared by the manufacturer: mL

Table 2. Changes in the content of energy and key nutrient, and in the proportion of ‘High in’ by food groups; comparison of two cross-sectional samples (pre-implementation period n = xxx, post-implementation period n = xxx).

|  | Content of energy [kcal], sugars [g], saturated fats [g] or sodium [mg] per 100 grams or mL of foods/beverages | | | | | | ‘High in’ [%] | |
| --- | --- | --- | --- | --- | --- | --- | --- | --- |
|  | Median | | 25^th^ percentile | | 75^th^ percentile | |  |  |
|  | PRE | POST | PRE | POST | PRE | POST | PRE | POST |
| Beverages  (N PRE=xx; N POST=xx) |  |  |  |  |  |  |  |  |
| Energy |  |  |  |  |  |  |  |  |
| Sugars |  |  |  |  |  |  |  |  |
| Saturated fats |  |  |  |  |  |  |  |  |
| Sodium |  |  |  |  |  |  |  |  |
| Savory baked products  (N PRE=xx; N POST=xx) |  |  |  |  |  |  |  |  |
| Energy |  |  |  |  |  |  |  |  |
| Sugars |  |  |  |  |  |  |  |  |
| Saturated fats |  |  |  |  |  |  |  |  |
| Sodium |  |  |  |  |  |  |  |  |
| Sweet baked products  (N PRE=xx; N POST=xx) |  |  |  |  |  |  |  |  |
| Energy |  |  |  |  |  |  |  |  |
| Sugars |  |  |  |  |  |  |  |  |
| Saturated fats |  |  |  |  |  |  |  |  |
| Sodium |  |  |  |  |  |  |  |  |
| Breakfast cereals  (N PRE=xx; N POST=xx) |  |  |  |  |  |  |  |  |
| Energy |  |  |  |  |  |  |  |  |
| Sugars |  |  |  |  |  |  |  |  |
| Saturated fats |  |  |  |  |  |  |  |  |
| Sodium |  |  |  |  |  |  |  |  |
| Candies & sweet confectionary  (N PRE=xx; N POST=xx) |  |  |  |  |  |  |  |  |
| Energy |  |  |  |  |  |  |  |  |
| Sugars |  |  |  |  |  |  |  |  |
| Saturated fats |  |  |  |  |  |  |  |  |
| Sodium |  |  |  |  |  |  |  |  |
| Sweet spreads  (N PRE=xx; N POST=xx) |  |  |  |  |  |  |  |  |
| Energy |  |  |  |  |  |  |  |  |
| Sugars |  |  |  |  |  |  |  |  |
| Saturated fats |  |  |  |  |  |  |  |  |
| Sodium |  |  |  |  |  |  |  |  |
| Yogurts, milks and milk-based drinks  (N PRE=xx; N POST=xxx) |  |  |  |  |  |  |  |  |
| Energy |  |  |  |  |  |  |  |  |
| Sugars |  |  |  |  |  |  |  |  |
| Saturated fats |  |  |  |  |  |  |  |  |
| Sodium |  |  |  |  |  |  |  |  |
| Dessert and ice-creams  (N PRE=xx; N POST=xx) |  |  |  |  |  |  |  |  |
| Energy |  |  |  |  |  |  |  |  |
| Sugars |  |  |  |  |  |  |  |  |
| Saturated fats |  |  |  |  |  |  |  |  |
| Sodium |  |  |  |  |  |  |  |  |
| Solid savory spreads & dressings  (N PRE=xx; N POST=xx) |  |  |  |  |  |  |  |  |
| Energy |  |  |  |  |  |  |  |  |
| Sugars |  |  |  |  |  |  |  |  |
| Saturated fats |  |  |  |  |  |  |  |  |
| Sodium |  |  |  |  |  |  |  |  |
| Liquid savory spreads & dressings  (N PRE=xx; N POST=xx) |  |  |  |  |  |  |  |  |
| Energy |  |  |  |  |  |  |  |  |
| Sugars |  |  |  |  |  |  |  |  |
| Saturated fats |  |  |  |  |  |  |  |  |
| Sodium |  |  |  |  |  |  |  |  |
| Dried fruits & snacks  (N PRE=xx; N POST=xx) |  |  |  |  |  |  |  |  |
| Energy |  |  |  |  |  |  |  |  |
| Sugars |  |  |  |  |  |  |  |  |
| Saturated fats |  |  |  |  |  |  |  |  |
| Sodium |  |  |  |  |  |  |  |  |
| Cheese  (N PRE=xx; N POST=xx) |  |  |  |  |  |  |  |  |
| Energy |  |  |  |  |  |  |  |  |
| Sugars |  |  |  |  |  |  |  |  |
| Saturated fats |  |  |  |  |  |  |  |  |
| Sodium |  |  |  |  |  |  |  |  |
| Ready to eat meals  (N PRE=xx; N POST=xx) |  |  |  |  |  |  |  |  |
| Energy |  |  |  |  |  |  |  |  |
| Sugars |  |  |  |  |  |  |  |  |
| Saturated fats |  |  |  |  |  |  |  |  |
| Sodium |  |  |  |  |  |  |  |  |
| NON-SAUSAGES MEAT PRODUCTS  (N PRE=xx; N POST=xx) |  |  |  |  |  |  |  |  |
| Energy |  |  |  |  |  |  |  |  |
| Sugars |  |  |  |  |  |  |  |  |
| Saturated fats |  |  |  |  |  |  |  |  |
| Sodium |  |  |  |  |  |  |  |  |
| Infant foods  (N PRE=xx; N POST=xx) |  |  |  |  |  |  |  |  |
| Energy |  |  |  |  |  |  |  |  |
| Sugars |  |  |  |  |  |  |  |  |
| Saturated fats |  |  |  |  |  |  |  |  |
| Sodium |  |  |  |  |  |  |  |  |
| Soups  (N PRE=xx; N POST=xx) |  |  |  |  |  |  |  |  |
| Energy |  |  |  |  |  |  |  |  |
| Sugars |  |  |  |  |  |  |  |  |
| Saturated fats |  |  |  |  |  |  |  |  |
| Sodium |  |  |  |  |  |  |  |  |
| Sausages  (N PRE=xx; N POST=xx) |  |  |  |  |  |  |  |  |
| Energy |  |  |  |  |  |  |  |  |
| Sugars |  |  |  |  |  |  |  |  |
| Saturated fats |  |  |  |  |  |  |  |  |
| Sodium |  |  |  |  |  |  |  |  |

Panel 1: Density curves of sugars content among food groups with relevant left-shifts, cross-sectional analysis.

|  |  |  |
| --- | --- | --- |
|  |  |  |
|  |  |  |
|  |  |  |
|  |  |  |
|  |  |  |

Panel 2: Density curves of sodium content among food groups with relevant left-shifts, cross-sectional analysis.

|  |  |  |
| --- | --- | --- |
|  |  |  |
|  |  |  |
|  |  |  |

Panel 3: Density curves of saturated fats content among food groups with relevant left-shifts, cross-sectional analysis.

|  |  |  |
| --- | --- | --- |
|  |  |  |
|  |  |  |
|  |  |  |

Panel 4: Density curves of energy content among food groups with relevant left-shifts, cross-sectional analysis.

|  |  |  |
| --- | --- | --- |
|  |  |  |
|  |  |  |
|  |  |  |

Supplementary Table 1. Cutoffs for defining regulated foods/ beverages in every phase

Supplementary Table 2. Type of foods classified in each food/beverage group

| Beverages |  |
| --- | --- |
| Sweet baked products |  |
| Ice cream & desserts |  |
| Ready-to-eat meals |  |
| Solid savory spreads |  |
| Breakfast cereals |  |
| Candies & sweet confectionary |  |
| Yogurts, milks & milk-based drinks |  |
| Sweet spreads |  |
| Savory baked products |  |
| Cheese |  |
| Dried fruits & snacks |  |
| Non-sausages meats |  |
| Baby foods |  |
| Soups |  |
| Sausages |  |

Supplementary Table 3. Changes in the content of energy and key nutrient, and in the proportion of ‘High in’ by food groups; comparison of a longitudinal subsample (n=xxx).

|  | Content of energy [kcal], sugars [g], saturated fats [g] or sodium [mg] per 100 grams or mL of foods/beverages | | | | | | ‘High in’ [%] | |
| --- | --- | --- | --- | --- | --- | --- | --- | --- |
|  | Median | | 25^th^ percentile | | 75^th^ percentile | |  |  |
|  | PRE | POST | PRE | POST | PRE | POST | PRE | POST |
| Beverages  (N =xx) |  |  |  |  |  |  |  |  |
| Energy |  |  |  |  |  |  |  |  |
| Sugars |  |  |  |  |  |  |  |  |
| Saturated fats |  |  |  |  |  |  |  |  |
| Sodium |  |  |  |  |  |  |  |  |
| Savory baked products  (N =xx) |  |  |  |  |  |  |  |  |
| Energy |  |  |  |  |  |  |  |  |
| Sugars |  |  |  |  |  |  |  |  |
| Saturated fats |  |  |  |  |  |  |  |  |
| Sodium |  |  |  |  |  |  |  |  |
| Sweet baked products  (N =xx) |  |  |  |  |  |  |  |  |
| Energy |  |  |  |  |  |  |  |  |
| Sugars |  |  |  |  |  |  |  |  |
| Saturated fats |  |  |  |  |  |  |  |  |
| Sodium |  |  |  |  |  |  |  |  |
| Breakfast cereals  (N =xx) |  |  |  |  |  |  |  |  |
| Energy |  |  |  |  |  |  |  |  |
| Sugars |  |  |  |  |  |  |  |  |
| Saturated fats |  |  |  |  |  |  |  |  |
| Sodium |  |  |  |  |  |  |  |  |
| Candies & sweet confectionary  (N =xx) |  |  |  |  |  |  |  |  |
| Energy |  |  |  |  |  |  |  |  |
| Sugars |  |  |  |  |  |  |  |  |
| Saturated fats |  |  |  |  |  |  |  |  |
| Sodium |  |  |  |  |  |  |  |  |
| Sweet spreads  (N =xx) |  |  |  |  |  |  |  |  |
| Energy |  |  |  |  |  |  |  |  |
| Sugars |  |  |  |  |  |  |  |  |
| Saturated fats |  |  |  |  |  |  |  |  |
| Sodium |  |  |  |  |  |  |  |  |
| Yogurts, milks and milk-based drinks  (N =xx) |  |  |  |  |  |  |  |  |
| Energy |  |  |  |  |  |  |  |  |
| Sugars |  |  |  |  |  |  |  |  |
| Saturated fats |  |  |  |  |  |  |  |  |
| Sodium |  |  |  |  |  |  |  |  |
| Dessert and ice-creams  (N =xx) |  |  |  |  |  |  |  |  |
| Energy |  |  |  |  |  |  |  |  |
| Sugars |  |  |  |  |  |  |  |  |
| Saturated fats |  |  |  |  |  |  |  |  |
| Sodium |  |  |  |  |  |  |  |  |
| Solid savory spreads & dressings  (N =xx) |  |  |  |  |  |  |  |  |
| Energy |  |  |  |  |  |  |  |  |
| Sugars |  |  |  |  |  |  |  |  |
| Saturated fats |  |  |  |  |  |  |  |  |
| Sodium |  |  |  |  |  |  |  |  |
| Liquid savory spreads & dressings  (N =xx) |  |  |  |  |  |  |  |  |
| Energy |  |  |  |  |  |  |  |  |
| Sugars |  |  |  |  |  |  |  |  |
| Saturated fats |  |  |  |  |  |  |  |  |
| Sodium |  |  |  |  |  |  |  |  |
| Dried fruits & snacks  (N =xx) |  |  |  |  |  |  |  |  |
| Energy |  |  |  |  |  |  |  |  |
| Sugars |  |  |  |  |  |  |  |  |
| Saturated fats |  |  |  |  |  |  |  |  |
| Sodium |  |  |  |  |  |  |  |  |
| Cheese  (N =xx) |  |  |  |  |  |  |  |  |
| Energy |  |  |  |  |  |  |  |  |
| Sugars |  |  |  |  |  |  |  |  |
| Saturated fats |  |  |  |  |  |  |  |  |
| Sodium |  |  |  |  |  |  |  |  |
| Ready to eat meals  (N =xx) |  |  |  |  |  |  |  |  |
| Energy |  |  |  |  |  |  |  |  |
| Sugars |  |  |  |  |  |  |  |  |
| Saturated fats |  |  |  |  |  |  |  |  |
| Sodium |  |  |  |  |  |  |  |  |
| NON-SAUSAGES MEAT PRODUCTS  (N =xx) |  |  |  |  |  |  |  |  |
| Energy |  |  |  |  |  |  |  |  |
| Sugars |  |  |  |  |  |  |  |  |
| Saturated fats |  |  |  |  |  |  |  |  |
| Sodium |  |  |  |  |  |  |  |  |
| Infant foods  (N =xx) |  |  |  |  |  |  |  |  |
| Energy |  |  |  |  |  |  |  |  |
| Sugars |  |  |  |  |  |  |  |  |
| Saturated fats |  |  |  |  |  |  |  |  |
| Sodium |  |  |  |  |  |  |  |  |
| Soups  (N =xx) |  |  |  |  |  |  |  |  |
| Energy |  |  |  |  |  |  |  |  |
| Sugars |  |  |  |  |  |  |  |  |
| Saturated fats |  |  |  |  |  |  |  |  |
| Sodium |  |  |  |  |  |  |  |  |
| Sausages  (N =xx) |  |  |  |  |  |  |  |  |
| Energy |  |  |  |  |  |  |  |  |
| Sugars |  |  |  |  |  |  |  |  |
| Saturated fats |  |  |  |  |  |  |  |  |
| Sodium |  |  |  |  |  |  |  |  |

Supplementary Panel 1: Density curves of sugars content among food groups with relevant left-shifts, longitudinal analysis.

|  |  |  |
| --- | --- | --- |
|  |  |  |
|  |  |  |
|  |  |  |
|  |  |  |
|  |  |  |

Supplementary Panel 2: Density curves of sodium content among food groups with relevant left-shifts, longitudinal analysis.

|  |  |  |
| --- | --- | --- |
|  |  |  |
|  |  |  |
|  |  |  |

Supplementary Panel 3: Density curves of saturated fats content among food groups with relevant left-shifts, longitudinal analysis.

|  |  |  |
| --- | --- | --- |
|  |  |  |
|  |  |  |
|  |  |  |

Supplementary Panel 4: Density curves of energy content among food groups with relevant left-shifts, longitudinal analysis.

|  |  |  |
| --- | --- | --- |
|  |  |  |
|  |  |  |
|  |  |  |
