# Supplementary material for: Changes in the amount of nutrient of packaged foods and beverages after the initial implementation of the Chilean Law of Food Labelling and Advertising: A nonexperimental prospective study
Source: PLoS Med. 2020 Jul 28;17(7):e1003220. doi: 10.1371/journal.pmed.1003220 (PMC7386631; doi:10.1371/journal.pmed.1003220)
Supplement: Attachment — Submitted filename: Response to reviewers, R1, 05.21.20.docx [file pmed.1003220.s007.docx]

REQUESTS FROM THE EDITORS:

**Abstract**
**Please combine methods and findings section to adhere to PLOS Medicine style**

Done.

**Please mention the data sources used**

Done, see lines 39 and 46.

**The last sentence of the methods and findings section must include a limitation of your study design/methodology**

Limitation included, see lines 60-61.

**Please provide 95% CI and p -values as needed when reporting numerical results or results of “significance”**

When needed, results now include 95% CI.

**Please replace the interpretation section with conclusions and begin this section with “our results show” or similar to avoid overreaching**

Edited, see line 62.

**Please remove funding information and provide this information in the funding section within the metadata**

Information removed and included in the right place, see lines 483-489.

**Data Availability
The Data Availability Statement (DAS) requires revision. For each data source used in your study:
a) If the data are freely or publicly available, note this and state the location of the data: within the paper, in Supporting Information files, or in a public repository (include the DOI or accession number).
b) If the data are owned by a third party but freely available upon request, please note this and state the owner of the data set and contact information for data requests (web or email address). Note that a study author cannot be the contact person for the data.
c) If the data are not freely available, please describe briefly the ethical, legal, or contractual restriction that prevents you from sharing it. Please also include an appropriate contact (web or email address) for inquiries (again, this cannot be a study author).**
Revised, see lines 474-481.

**On page 3 please replace the summary sub heading with “Abstract”**

Done.

**Author summary
At this stage, we ask that you include a short, non-technical Author Summary of your research to make findings accessible to a wide audience that includes both scientists and non-scientists. The Author Summary should immediately follow the Abstract in your revised manuscript. This text is subject to editorial change and should be distinct from the scientific abstract. Please see our author guidelines for more information:** [**https://journals.plos.org/plosmedicine/s/revising-your-manuscript#loc-author-summary**](https://journals.plos.org/plosmedicine/s/revising-your-manuscript#loc-author-summary)

Done.

Introduction
**Line 53 please revise to “In the last few decades …” or similar**

Done, see line 106.

**Please format all references according to Vancouver style and use square brackets within text**

Done.

**Lines 81-82 could you mention the years studied here to avoid ambiguity**

Done, see lines 135-136.

Methods
**Please mention names of all data source used**                                                                                                                                                          Done, see lines 143 and 174.

**Role of the funding source should be moved to the funding disclosure section in the article metadata**

Done, see lines 474-481.

**Results
Please ensure all numerical results are accompanied by p values and 95% CI when noting “significance” or the lack of it. For example Lines 200-201
Please provide exact p values, unless p<0.001**

Done when needed.

Discussion
**Please avoid assertions of primacy (Line 243) and add ” to our knowledge..”**

Done

**I note that potential confounders and effect modifiers were not assessed- should this be added as a limitation to the discussion?**

Done, see lines 442-443.

**Please ensure that the study is reported according to the STROBE guideline, and include the completed [STROBE or other] checklist as Supporting Information. When completing the checklist, please use section and paragraph numbers, rather than page numbers.**

STROBE checklists were included as supporting information.

**Please add the following statement, or similar, to the Methods: "This study is reported as per the Strengthening the Reporting of Observational Studies in Epidemiology STROBE guideline (S1 Checklist)."**

Statement included, see line 153.

**Please report your study according to the relevant guideline, which can be found here:** [**http://www.equator-network.org/**](http://www.equator-network.org/)

Done.


COMMENTS FROM THE REVIEWERS:

REVIEWER #1:

**This paper studies the changes in the proportion of food/beverages exceeding the cut-off of the Chilean Food Labelling and Advertising Law as well as the changes median content of energy and nutrients of these products after the implementation of this Law. It offers important contribution to literature as it provides the highly needed evidence on the potential effectiveness of labelling regulations on improving the nutritional quality of packaged foods and beverages. Attention to the following would improve the manuscript:
1.      A large part of the analysis mentions the relative percentage changes of the outcomes between the two periods. However, related figures are not given in tables 1 and 2, making it difficult for readers to follow. The analysis will therefore be clearer if additional columns are added for both samples to provide these percentage change figures.**

In the revised version we have divided the results derived from the cross-sectional and longitudinal analyses. Therefore, the proportion of ‘high in’ products are now presented in Tables 1 and 3. Both tables have now an additional column that displays the relative changes in the proportion of ‘high in’ for energy and each nutrient of concern, by food/beverage group.

**2.      One key finding of the paper is that the content of nutrients of most food groups decreased just below the cut-off from the visual analysis. However, this finding is not clearly explained in the paper, such as how to determine if the shift is just below the cut-off.  Given the large numbers of graphs presented, it would also be helpful to have some indications on which graphs display such left-shifts.**

We thank the reviewer’s comment and agree that this is a key finding that was not properly considered in the previous version. We have addressed this issue in the new version by changing the analysis to quantile regressions, and focusing specifically in significant changes that happened in the closest quartile to the cutoffs. In this way, we provide quantitative information to what is seen qualitatively in the Figures. We have completely restructured the results section and believe this finding is now much clearer.

**3.      Some food groups displayed a significant increase in saturated fats and energy after the implemented the law. The authors should provide some discussions on the potential reasons for these increases and their implications on the overall nutritional quality of packaged foods or beverages.**

We now discuss the potential reasons and implication of this finding, which is clear for saturated fats, but not for energy.

**4.      Line 131 - where is the sales data sourced from?**

Information added, see line 147.

**5.      The titles for figures 1 and 2 are missing.**

Titles and legends of figures are provided in the text, after the paragraph that first mention them, according to the Journal guidelines.

**6.      Line 224 - is the "5% change" an increase or a decrease?**

The results section has changed importantly in this new version and therefore this sentence is no longer available.

**7.      Line 247 - which nutrient is "the proportion of 'high-in' products" refereed to?**

This sentence is no longer available in the revised version.

REVIEWER #2:

**I confine my remarks to statistical aspects of this paper.  Unfortunately, I think that the wrong variable was analyzed.**

**When I first read the description of the project, I thought that, if I ran a food company, I would just lower all my numbers to just below the cutoffs. Then the product would not be marked as "high" in anything and more people than ever would buy my products.
Looking at Table 2 and the figures (which were excellent) confirms that that is what happened. The medians did not change much at all, but some of the top quartiles went down. In addition, the p values in this table seem to be off. Some tiny differences were highly sig. (e.g. sugars in desserts).**

**But I propose a fundamentally different analysis: I think quantile regression should be used, with time as the main iV and food group as a covariate. For the longitudinal analysis, a multilevel quantile model could be used.**

We really appreciate the statistical feedback provided. The current version uses quantile regression models (multilevel in the case of longitudinal analysis) for comparing the amount of energy and nutrient of concern, using quartiles of energy or nutrient of concern as dependent variable, and time (i.e., implementation period) as the independent variable. The 25^th^, 50^th^, and 75^th^ percentiles, now displayed in Tables 2 and 4, were predicted by such models. We did not include food group as covariate of the final models but rather one model per group because the food group interaction was significant.

Final models do not include food group as covariate given the significant interaction of such groups with the independent variable. As a matter of fact, it is expected that changes in the amount of energy and nutrient of concern are group-specific. For instance, improvement in the amount of sugars in sweet products, or more pronounced changes in the amount of sodium in savory groups.

REVIEWER #3:

**Changes on the nutrient content of packaged foods and beverages after the initial implementation of the Chilean Law of Food Labelling and Advertising: a nonexperimental prospective study.**

**The impact of regulation of foods is currently of great public health interest and this piece of work examining the introduction of mandatory labelling on processed foods and drinks is therefore welcome. However, I have some concerns over the analysis and manuscript. The chosen analyses methods could be stronger as the results presented show simply pre- and post-law differences that fail to consider existing trends in nutrient content. It is possible that these differences would have been seen if the law had not come into effect not least because the Chilean government signalled its intent to use regulation through the modification of their tax on SSBs. This should be clearly stated in the limitations section.**

We agree with the reviewer that the design of the study does not allow assuming a causal association between the implementation of the regulation and the observed changes in energy and nutrient of concern. We have highlighted this limitation by including the design (i.e., non-experimental prospective study) in the title of the article, and discussing this as the main limitation in the specific section and in the abstract. In terms of previous trend it is important to note that we have confirmed that previous to the implementation of the law there was no relevant change in nutrient information of packaged foods (Kanter R. Ob Reviews 2019). Moreover, we believe that the fact that in most cases foods/beverages shifted just below the regulatory cutoffs makes it more likely to assume that the observed changes could be the result of the law implementation. In the current version we have included quantile regression analyses that allow us to statistically test these changes.

**Title.
Change "on" to "in" i.e. "Changes in the nutrient content of packaged foods and beverages after the initial implementation of the Chilean Law of Food Labelling and Advertising: a nonexperimental prospective study".**

Done.

Abstract.
**Background
The first sentence of the background section doesn't make sense; suggest changing to something like: "In June 2016, the Chilean Food Labelling and Advertising Law that mandated front-of-package warning labels and marketing restrictions for unhealthy foods/beverages was implemented.**

Edited, see lines 35-37.

**Methods
Sometimes the time periods are referred to as T0 sometimes as T0 please be consistent.**

In the current version, we have used T0 (and T) throughout the entire document, including Tables, Figures and legends.

**Although later specified it is not immediately clear what T0: Jan-Feb 2015/2016 refers to. Suggest clarifying this earlier. Perhaps reword the first sentence in the methods to state that data collection was carried out at two time periods.**

Done, see lines 40-41.

**N= is written twice - remove one.**

Done.

**There is a semicolon before N= in T1 but not in T0, change to be consistent.**

Done.

**First sentence of the methods should include a hyphen after pre.**

Done.

**The first sentence is too long. Suggest starting a new sentence after T1. As it stands it doesn't make sense particularly. Change to something like "A longitudinal subsample was also studied to explore changes in the median content of energy and nutrients of concern (total sugars, saturated fats and sodium, per 100g/100mL) and in the proportion of products with energy and nutrients exceeding the cutoffs of the Law (i.e., 'high in' products).**

Edited as suggested.

**Findings
"High in" has not been defined, suggest changing to "product high in unhealthy nutrients".**

This part of the text is no longer available in the current version.

**"Significant decrease" should be changed to "Significant decreases".**

This part of the text is no longer available in the current version.

**Change the latter part of the penultimate sentence to "whereas changes in the amount of energy and saturated fats were uncommon." writing "content of energy" suggests a binary state of energy or no energy - which is unlikely to occur.**

In this new version, we have replaced “content” by “changes” throughout the entire document, including Tables, Figures and legends.

**Interpretation.
Remove the final "s" from "sugars".**

We prefer maintaining the final “s” for “sugars” given it groups a set of mono and disaccharides. Actually, the FOP warning label in Chile reads: “high in sugars”.

**Author Affiliations**

**What are the numbers in the author affiliations? Are they postcodes / zip codes?**

They are zip codes, the information is now added.

**Line 21 why is "Stefanie Vandevijvere Senior Public Health Nutrition Scientist" written?**

Removed.

**The author affiliation for institute 1 does not match that of the corresponding author.**

Edited.

**Introduction
Line 53 "In the last decades is too ambiguous, if this manuscript is being read in 20 years time it will not make sense. Please be more precise e.g. "From 1990-2020, packaged foods..."**

Edited according to the reviewer’s suggestion.

**Personally I find the use of ultra processed foods unhelpful. That is, the implicit suggestion that simply processing the food makes it worse than not processing the food. The law referred to in this manuscript addresses nutrients rather than the number of processes that are required to produce the final food. The second sentence states that it is the high content of energy and nutrients linked to NCDs that is the issue. I am aware of the two positions of investigating diet either by examining total foods or looking at nutrient composition. But the ultra-processing part feels too poorly defined. Sure sausages and processed meat are linked to increased risk of NCDs but then this law focuses on the nutrient composition. Line 59 further discusses improving nutritional quality by reducing nutrients of concern rather making foods less processed. In short could you not write ultra-processed foods in the first sentence of the introduction as you appear to be interested in nutrients and should therefore write that the package foods and beverages contain more nutrients of concern rather than are "processed".**

We have removed “ultraprocessed foods” given that we agree with the reviewer that this concept is not particularly relevant in this report. However, we would like to briefly indicate why we used it in the previous version. The Chilean regulation target foods/beverages based on their amount of energy or nutrient of concern, however it only applies to foods/beverages that have been added with ingredients that increase the natural amount of nutrients of concern and thus. We believe that the Chile regulation it does consider in some extent the idea of processing.

**Line 60 change content to level or amount. Again content is like contains - does it contain nutrients of concern? Yes - it has energy.**

As previously commented, we use “amount” in the current version of the manuscript.

**Line 61. I think we have been interested in the nutritional quality of packaged foods for longer than "since the 2000s".**

This sentence has been edited, please see line 114.

**Line 65 change contents to content.**

Done.

**Line 65 change i.e. to e.g. as these are examples of possible strategies rather than the exhaustive list of all possible strategies implied with i.e.. Also include "adding" (or something similar) before "upper limits for sodium content" as it can be read as banning trans fats or banning upper limits for sodium content.**

Done, see line 119.

**Line 67 "However, as most of these strategies are voluntary the impact on the nutritional quality of the overall food supply has been limited". I disagree. There are lots of mandatory strategies that incentivise reformulation such as mandatory back of pack labelling, for example, fortification of wheat with calcium, folate and iodine fortification, SSB and fat taxes and VAT/GST. Similarly there are restrictions on more classical food safety grounds like the lead and arsenic content of foods for example. Recommend that this sentence is amended to make the point I think you are trying to make which is that without mandating these strategies industry is less likely to do what is wanted and therefore government intervention is required.**

The sentence has been re-written in order to consider the reviewer’s suggestion, please see line 122.

**Line 70 feels a bit cumbersome to refer to the law by its full title every time. Could you use an abbreviation?**

In the current version of the manuscript we use “law”.

**Line 73 I suspect the implementation was not agreed in a staggered way rather the law was to be implemented in a staggered way, please amend accordingly.**

Done, see line 127.

**Line 75 sentence starting "Such products..." which products? This seems out of context or at least needs changing to fit.**

This part of the text is no longer available in the current version.

**Line 76 Not sure what the "Chilean experience" is. Should this be something like "The Chilean government has introduced a wide range of strategies to improve public health and tackle childhood obesity that combines a FOP label in combination with child obesity prevention strategies..."**

Edited according to the reviewer’s suggestion.

**Line 76 child obesity should not be hyphenated.**

Removed.

**Line 79 sentence starting "Therefore, in..." this could do with tightening up. Suggest something like "Therefore, in the current study we aimed to study changes in the proportion of 'high in' products and changes in energy and nutrients of concern in packaged foods and beverages, before and after the initial implementation (i.e., <1 year of the first phase cutoffs) of the Chilean Law.**

Done, see lines 134-136.

**Line 81 1 year does not need hyphenation.**

Removed.

**Methods**
**Line 87 pre does need a hyphen**

Done.

**Line 87 as above T0 and T1 have a comma before N=... Please be consistent.**

Done.

**Line 87 the colon after T0 is subscripted it should not be.**

Done.

**Line 89 was the Law only implemented in Santiago or are you only looking at Santiago? Clarify the interest in Santiago ideally give context of Santiago with the rest of Chile.**

Done, see line 143.

**Line 89 new sentence after Chile.**

This part of the text is no longer available in the current version.

**Line 90 hyphen after pre**

Done.

**Line 94 are you interested in ingredients? Is the amount of each ingredient given on the label otherwise you won't be able to see reductions in the amount other than perhaps a change in the order of the ingredient (any way I suspect without amounts/percentages it's going to be inexact).**

The Chilean regulation requires that producers report amounts per 100g/100 ml of energy, total fats (subtypes), carbohydrates, protein, sodium and any other nutrient related to a health claim (i.e. fiber if the product claims is high in fiber). It does not require reporting the amount of ingredients. However, we are interested in the ingredient list because we can check in this list whether the food product has addition of energy or any nutrient of concern and therefore, is subject to the law (which is explained in lines 225-226).

**Line 94 why were you taking photographs of products before the law was introduced? Was this work for something else or were you aware that the law was going to be introduced? If it is the former then it's not a big deal and perhaps just include a sentence addressing this if it's the latter, that is, the authors (and manufacturers) were aware in 2015 the law was going to be introduced then your baseline sample could be biased.**

We collected data on the food supply because we were participating in an international network (INFORMAS) interested in characterizing the food environment including the quality of the food supply. However, we would like to note that the decree indicating the implementation details of the law was published in June 2015 (12 month before the initial implementation). We have the same concern as the reviewer and we conduct and analyze comparing the nutrient content of packaged foods in 2015 and 2016 and we observed no anticipatory reformulation (Kanter R, Ob Reviews 2019) so we are confident of the validity of the baseline data we are using.

**Line 96 This had to be read a couple of times to get the meaning perhaps "(one supermarket from each of the 6 major chains in Chile)"**

Edited according to reviewer’s suggestions.

**Line 96 could you give an indication of the market share of these 6 chains for those unfamiliar with Chile?**

Done, see lines 159-160.

**Line 96 are the 3 candy distributors localised to the high income areas? Could you add more clarity to this as I can only think of shops that distribute to a local area and that any distributor would be at a wider geographic area.**

Candy distributors were located in high income areas; they were included given the broader spectrum of candies and sweet confectioneries compared with supermarkets and local shops. Explanation was added in lines 161-163.

**Line 99 could you define packaged foods? Is that essentially all foods? Does it include packaged fruit and vegetables?**

Packaged foods refer to those foods packaged prior to being selected or sold by consumers, and thus, excludes foods that are packaged only upon request, as bulk foods or foods asked at the counter. An explanation has been added, see lines 165-167.

**Line 99 did you explore alternatives to visiting stores such as manufacturer or supermarket websites? The wayback machine can be useful for retrospective website access.**

We did explore this option; however, In Chile manufacturers and supermarkets do not include updated nutrient content of food products.

**Line 105 did you carry out any validation of the data, for example we saw a temporal changes in product labelling when manufacturers went from providing nutritional information per 100g to providing nutritional information as made up with semi-skimmed milk. This resulted in dramatic reduction in sugar and increases in protein for the same unreformulated product. Possible checks could be to examine the atwater factors (e.g. fat = 9kcal per 1g) and see if based on the macronutrient content energy is as expected.**

We did validate our results as mentioned by the reviewer. We have now added this information in the manuscript, see lines 172-176.

**Line 116 i.e. should be changed to e.g.**

Done.

**Also the list provided (nuggets and others) does not provide a lot of clarity, simply writing non-sausage meat products seems like a rather odd classification where I am unsure what is special about sausages but at least seems easy to understand whereas the examples of nuggets and others make it more not less ambiguous.**

The example has been removed.

**Line 121 I am unclear why you included two years of data in the baseline sample. The issue with this is - and I'll come to this in the analysis section - is that when you carry out a simple pre post study like this you don't know why the changes occured. They may be due to the law but they may simply reflect on-going reformulation in the food market and that these differences would have appeared if the law had not come into effect. Comparing data from 2015 to 2017 means that the changes are more likely to have come about due to the general reformulation towards healthy products. In short could you explain the benefit for including two years of baseline data as it is not immediately apparent.**

We pooled 2015 and 2016 data in order to have a greater baseline dataset because some food groups were underrepresented in the 2015 data set (the information that year was collected under a different project). We have confirmed that there were no significant changes on the food supply between 2015 and 2016 (Kanter R. Ob Reviews 2019) so we are confident that the data of the two years can be pooled.

**If the product was available in 2015 and 2017 but not in 2016 then either it was not captured in 2016 or it was withdrawn and relaunched. If it is the former then it casts doubt on the completeness of the sample.**

Only 30% of our baseline dataset was collected only in 2015 and not collected in 2016. The primary reason is due to the fact that 2016 data collection was defined as a round of data collection that would complete the 2015 data and therefore, was more oriented to categories incompletely or not collected in the 2015 round. We agree with the reviewer we cannot fully exclude issues of sample collection; however, the fact that longitudinal analyses provide similar results than the ones of the cross-sectional analyses make us confident of the validity of our sample collection.

**Also why not do two years post implementation i.e. 2018? Including two years pre-implementation data seems to confuse things, raise questions and add little. It feels like the 2015 data have been included simply because they were collected and are available.**

We did not include the year 2018 because in June 2018 started the second stage of the regulation and therefore, it would not be appropriate to pool the 2017-2018 data.

**Line 123 (i.e., 2016 items were included) suggested rewriting to something like "only items collected in 2016 were included" to remove ambiguity**

Done, see lines 197-198.

**Line 129 As I understand it you are only interested in individual products with greater than 1% market share. What percentage of products have less than <1% market share? Having 100 products on the market means that all of these (except for a couple) would be excluded - or have I missed something?**

In order to obtain more meaningful data, we eliminated from the analysis products with small market share within a specific food group (i.e., those which sales represents less that 1% of the specific food category, according to Euromonitor data). Thus, only products with sales >= 1% of their category were included. About 50% of T0 and T1 samples were removed because of this reason. This is better explained in this new version, see lines 205-207, and Fig 1.

**Line 145-146 line spacing looks smaller than the remainder of the document**

Edited.

**Line 149 food densities, more technically known as specific gravity.**

Edited.

**Line 152 Would help if the two analyses that are presented (i.e. cross-sectional and longitudinal) had their own subsections**

We thank the reviewer for this suggestion, we have separated both analyses in this new version, what is reflected in the text, Tables and Figures.

**Line 161 law should be lower case.**

Edited where suggested and throughout the text.

**Line 163 hypen for pre**

Done.

**Line 166 "distributions were non-parametric". This is incorrect and should be changed. I suspect what is meant here is that the distributions were not normally distributed and therefore non-parametric tests were used. Distributions have parameters, tests that do not rely on these parameters are non-parametric.**

Edited.

**Line 167 Was there any adjustment for multiple testing? If one component of the food changes then the others are more likely to do so. Similarly manufacturers may make the decision to reformulate across their entire product range and therefore these tests are not independent and this should be addressed accordingly - typically be lowering the alpha threshold.**

We agree with the comment of the reviewer. In this version we have followed the statistical reviewer advise and run quantile regression. We were unclear on how to penalize models for multiple testing in these regressions, but we have mentioned in the limitation section that we did not did a formal correction and highlighted that we should trust more changes that have p<0.01.

**Line 167 Analyses should not have a capital letter as the second word in the subtitles does not anywhere else.**

Edited.

**Line 174 insert "at" between available and each**.

Done.

**Line 174 remove space after /**

Done.

**Line 175 see earlier point. For food groups with 482 products the market share for the majority will be <1% whereas the group with only 69 will have a greater number of products with 1+% market share (probably) therefore the results will be biased towards categories with fewer groups.**

We have better explained the exclusion due to the irrelevance in the market share in this new version; we hope to have clarified this issue.

**Line 176 should it be "in" before "the standard..."?**

Edited.

**Line 180 should be analyses**

Edited.

**Line 184182 error**

Edited.

**Results**
**Is it possible to have a summary of all changes carried out? For example if 5 groups have shown a decline but 5 have increased then there may be no overall benefit. Or perhaps not all food groups are equal as small changes in foods that are highly consumed may have a greater impact on health than large changes in foods that are rarely consumed. Similarly is there a hierarchy in the changes that were observed that is: are changes in energy more important than changes in sodium for example?**

We agree with the reviewer that we need to provide some context to the changes observed. First, we have clarified that we only included foods that account for >1% of the share of its food category; then, in the discussion we have added the amount of sugars and sodium intake that represent each of the food categories in which we observe changes as well as stating that the ultimate impact of the observed food reformulation should be observed with dietary intake follow-ups.

**Line 187 would prefer % after each percentage value e.g. 51% to 44%.**

Done.

**Line 188 "left columns" is unclear please use column numbers or change.**

The text is no longer available in this new version.

**Line 189 can you provide confidence intervals?**

The exact text is no longer available in this new version, but we have provided confidence intervals when needed in the new version.

**Line 206 change left column to figures on the left or something similar.**

This is not needed in this new version since cross-sectional and longitudinal results are displayed in different figures.

**Line 209 see above. It is unlikely that foods will be reformulated for a single nutrient. If reformulation occurs it is likely to change energy and nutrients.**

We agree with the reviewer that this is the case of sugars or saturated fats and energy (although some replacement within those nutrients is also possible). As previously said, we have followed the statistical reviewer advise and run quantile regression. We were unclear on how to penalize models for multiple testing in these regressions, but we have mentioned in the limitation section that we did not did a formal correction and highlighted that we should trust more changes that have p<0.01.

**Line 214 should be "Changes in".**

Done.

**Line 214 It is unclear what is meant by this sentence, changes...were observed for products in which there was no...change.**

The text is no longer available in this new version.

**Line 219 please write Figures in full each time.**

According to author guidelines, we have maintained “Fig” instead of the full word.

**Line 220 remove "overall".**

Done.

**Line 228 change "on" and "among" to "in" and as appropriate elsewhere.**

Done.

**Line 229 change "among" to "in" and as appropriate elsewhere.**

Done.

**Line 223 change to (by between 1% and 6%, depending on the food group)**

Done.

**Line 214237 error.**

Edited.

**Discussion**
**Line 243 change to "This is the first study to evaluate changes..."**

Done.

**Line 247 what is meant by "relative" decrease?**

The text is no longer available in this new version.

**Line 250 this is written to appear that the groups were the driver of the change whereas the groups are passive and had change done to them.**

Edited.

**Line 255 "significant improvements were seen".**

Done.

**Line 257remove has been described (or change to have)**

Done.

**Line 259 change "supply" to "suppliers" if appropriate.**

Done.

**Line 259 change "...menus of fast..." to "menus in fast..."**

Done.

**Line 261 remove "the distribution of".**

Done.

**Line 262 remove comma after USA.**

Done.

**Line 262 change first "on" to "of" second "on" to "in".**

Done.

**Line 263 references after the end of the sentence.**

Done

**Line 268 "given a previous report..." this sentence is not clear please rewrite.**

We have re-written this sentence. See xxx.

**Line 271 add "the" before "healthy school food environment".**

Done.

**Line 272 agree that changes can be brought about with incentives however this cannot be continued indefinitely.**

We agree with the reviewer; we have change this sentence stating that actions should be made to ensure that industry make these changes.

**Line 277 change "in their way to implement" to "on their way to implementing".**

Done.

**Line 280 remove "between".**

Done.

**Line 281 change "2015 Chilean packaged food supply in Chile" to "packaged food supply in Chile in 2015" or something similar.**

Done.

**Line 284 change "have" to "has".**

Done.

**References
Some of the journals are abbreviated and some are not. For example Obesity Reviews is given in full and as Obes Rev**

**American journal of public health requires capital letters for all words.**

**Some article titles have capital letters for each word some do not.**

**Page numbers are not included for all articles e.g. Ref 2 includes page numbers Ref 6 does not.
Page number for Ref 1 should read 10-19 not 10-9**

**Refs 7, 30, 32 only have 1 page number (2/836/65)**

We appreciate this careful review, we have edited reference according to Journal guidelines, editors and the reviewer’s request.

**Table 1**

**Remove extra line space in Savory baked products - High in Sodium and Sausages - High in Sodium.**

All tables have importantly changed in this revised version of the manuscript, considering all the reviewer’s comments.

**Table 2**

**Remove space between 100 and mL**

**footnote "n" is lower case - change to upper case for consistency.**

**Why are there sometimes [ and sometimes (?**

**Sometimes there is a space and a long dash sometimes no space and hyphen. Please be consistent.
Please use consistent number of decimal places.**

All tables have importantly changed in this revised version of the manuscript, considering all the reviewer’s comments.

**Figure 1**

**Not sure why the title is all in capitals,**

**Subsample and exclusions are spelled incorrectly.**

**Sometimes the thousands digits are seperated by a comma sometimes they are not - please be consistent.**

**Some of the lines do not join into the middle of the boxes, sometimes there are spaces between the boxes and the lines.**

**Recommend that the final boxes are aligned.**

**Sometimes there are spaces after the = sign, sometimes there are not - please be consistent.
The first horizontal line looks slightly skewed, that is not perfectly horizontal as it has a step in it.**

**Sometimes the description is given before the N sometimes after - please be consistent.
Change the boxes with N T0=10,081 and N T1=8,563 to match the others by changing the position of the N i.e. T0: N=10,081 ...**

**In the final boxes T0 and T1 are not sub(sub)scripted**

We have reviewed Figure 1 and incorporated all suggestions.

**Figure 2**

**Please include all plots (Energy, Sugar, Sat Fat, Sodium) for each category
n= has become lower case here, please use the same case throughout.**

**Use T0 and T1 rather than Pre and Post**

**Include a footnote to state why the numbers (n) aren't the same for energy and sugars
Why do the density curves begin at different points <0?**

**Remove space between 100 and mL for consistency.**

**The resolution of these plots is not great - can you change to vectorised images?
The contrast between the green and blue is not great - consider more contrasting colours though maybe this would be improved with resolution changes.
Consider shading the area under the plot**

**Why is there white space above some of the curves in some plots and not others? Ideally the same scale would be used but failing that at least make them look similar.
Same with the x axis - some curves fill the x axis whereas others have lots of space.
Breakfast cereals - it appears that some breakfast cereals have (close to) 0kcal of energy. Is this correct?**

**Should be non-sausage meat products throughout the manuscript.**

**Use "Cheese" or "Cheeses" throughout the manuscript for consistency.**

**Where are the plots for Ready-to-eat meals?**

We really appreciate the detailed review of this Figure. We have incorporated all the suggestions in this reviewed version, along with the fulfillment of the Journal’s guidelines. The former Figure 2 has become Figure 2 and 3 in this new version.

**Supplementary Table 1**

**Please make line spacing the same for both solids and liquids.**

**Why are square brackets used here for the first time - please be consistent.
No spaces after 100**

Done.

**Supplementary Table 2**

**The foods seem to be ordered by random - could you make it alphabetical or are they by number of products?**

**These categories need lots of revision.**

**Unclear what the difference between "flavored water with sugar" and "aromatized/flavored waters with sugar" is.**

**Are "fantasy beverages" a Chilean product?**

**The "other" group should go at the end of the list to catch those products not categorised previously.
There are three categories for "fantasy beverages" with sugar, without sugar and other. The first two categories cover all possible options - what goes in the final category?
What is the difference between "100% fruit juices" and "100% fruit juices (no added ingredients"? Surely if it is 100% fruit juice then it is only fruit juice and has nothing else.
Flavoured water with sugar is repeated but spelled differently.
What are milk drinks? That seems to encompass all of the subsequent categories.
Change "skim" to "skimmed" for consistency.**

**Yogurt is in the Milk and milk-based drinks category and in the yogurt category.
Remove spaces around /.**

**Light or diet yogurt includes light or diet yogurt with fruits and/or nuts.
Breakfast cereals - change to "dry fruit cereal bars".**

**The three cereals categories seem very specific i.e. balls, honey stars and flakes. Are there a lot of honey stars cereals in Chile?**

**Dessert & ice cream - change to vegetable chips (remove s)
Candies & sweet confectionery - why are nuts in here?**

**Remove s from popcorns**

**Confectionery is spelled two ways - confectionery and confectionary.
Why are chocolate chips in sweet spreads not "Candies and sweet confectionery"?
What is "glazed"?**

**Jam (all kinds) includes the other jam category.**

**What is "other products used for pastry"?**

**Savory baked products - two spaces after "sopaipillas,"**

**What is the difference between "packaged white bread" and "packaged white bread loaf"?
What is the difference between " packaged whole wheat bread loaf (regular, light or diet)" and "packaged whole wheat bread loaf (all kinds)"?**

**"Corn torillas" is repeated.**

**Should be "vegetable broth" not "vegetables broth".**

**"Other dressings" and "other dressings for salads" do not appear to be mutually exclusive - please amend.**

**Savory spreads - remove spaces after / to be consistent with elsewhere.
What is the difference between "fresh cheeses and light cheeses" and "fresh cheeses"?
Why is bacon and ham in the sausage category?**

**Sweet baked products: are bizcochos and biscochos different things or is that a typo?
"Frozen breaded meat" and "frozen marinated or seasoned meat" are written twice
What is the difference between "nuggets (chicken or turkey)" and "nuggets".
Unclear what the difference between "Soups for one or other type of instant soups" and "instant soups" is.**

We have importantly reviewed this Table according to the reviewer’s suggestions.

**Supplementary Table 3**

**The first sentence isn't clear, "outlier" should be changed to "outliers" and the final part "considered unlikely to happen for the specific group were omitted" needs rewriting.
Would be helpful to state that these values are per 100g or serving or whatever the units are
N is lower case unlike in the majority of the document - please be consistent**

**ii should be "Sugar" not "sugars".**

**iii should be "fat" not "fats".**

We have edited the Table according to the reviewer’s suggestions.
