# Supplementary material for: Changes in the amount of nutrient of packaged foods and beverages after the initial implementation of the Chilean Law of Food Labelling and Advertising: A nonexperimental prospective study
Source: PLoS Med. 2020 Jul 28;17(7):e1003220. doi: 10.1371/journal.pmed.1003220 (PMC7386631; doi:10.1371/journal.pmed.1003220)
Supplement: Attachment — Submitted filename: Response to reviewers, R2.docx [file pmed.1003220.s008.docx]

We thank the Academic Editor and Reviewers for this new round of reviews that -again- have helped us to improve importantly the new version of the manuscript. We here provide responses to every comment.

**REQUESTS FROM EDITORS**

**Comments from AE:**
**I think to understand the impact of a labeling process, one needs to look carefully at the specific distributions and where the line is set for something to be declared "high". In the case of sugars in milk drinks, for example, the distribution is fairly flat and the line for being declared high is in the middle of the distribution. There is a clear shift in the distribution post-introduction of labeling. Other distributions are fundamentally different and the line of where "high" is are sometimes way above the top end of the distribution pre-labeling. It is no wonder that there is little impact. In addition to making the changes that have been proposed in this paper, I would rather hope that any resubmission might highlight more clearly some things like the above, which are messages that don't seem to be coming through very strongly at the moment.**

We thank the Academic Editor for this important comment. We agree with him/her that this message was not present in the former version and was important to include it. We have added a description of results along these lines in the abstract (see lines 44-45), author summary (see lines 82-83), results (see lines 279-284), and discussion (see lines 575-578). The current discussion also includes a reflection on the implication of the cutoffs for promoting reformulation (see lines 597-613).

**Please remove the original submission file from the submission system as can erroneously be used to generate the full submission PDF by the system.**

Done.

**In the abstract- its not clear what you mean by a longitudinal subsample that was also analysed. Please clarify and revise as needed.**

Done, see lines 39-40.

**Line 114- perhaps you can revise to 20th century using roman numerals?**

Done, see line 112.

**Line 143- I think you mean 30% of the population in Chile lives. Please revise.**

Done.

**Did your study have a prospective protocol or analysis plan? Please state this (either way) early in the Methods section.
a) If a prospective analysis plan (from your funding proposal, IRB or other ethics committee submission, study protocol, or other planning document written before analyzing the data) was used in designing the study, please include the relevant prospectively written document with your revised manuscript as a Supporting Information file to be published alongside your study, and cite it in the Methods section. A legend for this file should be included at the end of your manuscript.
b) If no such document exists, please make sure that the Methods section transparently describes when analyses were planned, and when/why any data-driven changes to analyses took place.
c) In either case, changes in the analysis-- including those made in response to peer review comments-- should be identified as such in the Methods section of the paper, with rationale.**

We did have a statistical analysis plan before starting the analyses that was included in a concept note that we circulated with the study coordination board; however, quantile regression analyses were included in a later version as a response to the suggestion made by the statistical advisor of the journal. As requested, we have provided this information on the methods section and in the current submission we have included the concept note with the corresponding legends.

**Please remove page numbers and line numbers from STROBE checklists provided as these are likely to change. Please use paragraphs/sections instead.**

Done.

**The financial information can be removed from the word document as it is automatically pulled from the article meta-data. Please only provide the information in the financial disclosure section- Same goes for the data availability information, which can be provided in the data statement.**

Both financial information and the data statement have been removed from the word document and are now presented only in the article meta-data.

**In the discussion you say that your results suggest that food and beverage reformulation occurred rapidly. I’m not sure if this can be directly linked, as you have noted in the limitations section, so I recommend removing this sentence and carefully toning down the discussion to avoid overstating your conclusions.**

We have revised the discussion according to Editor’s advice.

**Please add a sentence in the Competing Interests statement to
acknowledge that Barry Popkin is an Academic Editor for PLOS Medicine.**

We have now reported Barry Popkin’s position in the corresponding section.

**REVIEWER #1**

**The authors have addressed my previous comments. The
quantile analysis has strengthened the paper and made it easier for
readers to understand the changes occurred around the cut-off.**

We thank the reviewer for this comment and agree the quantile analysis has considerably improved the manuscript.

**Only a
few minor comments left:
Line 114 what is XX?**

This has been changed by 20^th^ given it was meant to indicate the 20^th^ century.

**Line 167 "small packages where not collected" It is unclear what it is meant.**

This has been changed by “only the largest package was collected”.

**Line 257 How are "food/beverage groups" controlled/ assessed in the
quantile regression?**

In the beginning, quantile regressions for the overall sample were performed, considering covariates food/beverage groups and the interaction of such groups with time. Given the interaction was significant in every model (i.e. energy and nutrient of concern), analyses were done stratifying by food groups. We thank the reviewer for this comment as this was not clearly stated in methods, which is now corrected (see 256-259).

**REVIEWER #2**

**The authors have addressed my concerns and I now
recommend publication**.
We thank again the reviewer for his valuable statistical advice.

**REVIEWER #3**

**The manuscript is much improved. I have a few, minor comments.**

We thank the reviewer for his/her two careful and detailed reviews that have helped improving the current version of the manuscript.

**Line 76 - school sales at school. Seems like the first "school" is not required**

Edition done, see line 71.

**Line 114 - add in years instead XX**

This has been changed to 20^th^ according to the Editor’s suggestion, see line 112.

**Line 165 - no comma after i.e.**

We have edited this throughout the text.

**Line 166 - add for after asked or change asked to chosen.**

Done, see line 165.

**Line 167 where should be were.**

Done, see line 164.

**Line 173 no comma after i.e.**

We have edited this throughout the text.

**Line 274 two spaces after all.**

Done.

**Line 273 remove hyphen from sweet baked.**

Done.

**Line 337 associated with a decrease not associated to.**

Done, see line 446.

**Line 380 reports of improvement not to improvement.**

Done, see line 593.

**Line 403 change in to on.**

Done, see line 638.

**Line 412 add reference for the National Dietary Survey.**

Done, see line 651.

**Line 405 put such in front of as.**

Done, see line 640.

**Lines 410-415 417-424 combine in to single paragraph.**

Done.

**Line 446 remove the.**

Removed.

**Line 642/670/736 Cutoff corresponds - add s.**

Done.

**Line 643/671/737 comma after i.e.**

We have edited this throughout the text.

**Line 681/747 McNemar tests**

Done.

**Table 1 - What do the confidence intervals represent? Should this be interpreted as, for example, 26% of beverages were "high in" any category in T0? If so this doesn't require a confidence interval as it is a single value with no variance. Also some of the point estimates do not lie exactly between the confidence intervals, e.g. sweet baked products - high in sugars 95 (90-97).**

We have preferred to provide some uncertainty boundaries to our estimates because we can’t be 100% certain that we collected the universe of products; confidence intervals are not symmetric because we assumed a binomial distribution for their estimation.

**Table 2
Savoury snacks sodium T1 - remove bracket**

Done.

**Sometimes one or two decimal places are given sometimes there are none.**

We have used 2 decimals for all values from Tables 2 and 4.

**Desserts & ice creams sugars - for p25 and p50 the values at T0 and T1 are the same and the p-value is 1 whereas for p75 T0 and T1 are the same but the p-value is 0.30. Is this because of rounding?**

Differences are clearer now that 2 decimals are displayed.

**Table 3
Beverages Any 'High in' the dash is different to the others.**

We were not able to identify this particular problem, but in the current version we have carefully reviewed the format of Table 3

**Ready-to-eat meals, please check n as elsewhere it is 109.**

Done.

**Table 4
Candies & sweet confectionary is used, whereas confectioneries is used
elsewhere.**

The change was made.

**Savory spread sugars p25 T0 = 0, T1 = 0 but p!= 1 please add decimal
places to explain.**

Done.

**Fig 1
Include a line linking total products collected to products collected in 2015**

Done

**Some of the arrows are not exactly horizontal**

Changes made.

**Left hand side exclusion box
Change n to lower case
change semi-colon to comma for consistency**

Both changes done.

**Right hand side exclusion box - remove colon and change semi-colon to comma**

Done

**Fig 2
Unclear why the x axis start below 0. Are there negative numbers given
or is this the default plot?**

Density graphs generate density plots around the exact estimate. Thus, if the observed variable has values of zero or close to zero then the density plot may include values to the left of zero.

**Kcal does not begin with a capital K elsewhere in the document**

This has been edited in all figures.

**In some instances the n provided in the plot does not match that given
in table S2, for example Fig 2C n for yogurt is 181 whereas Table S2
states 184. Similarly n for cheeses = 60 and 61 in S2, Fig 2M T0 n=242
Table S2 states 243. This may be due to some products not providing
all information if this is the case then perhaps provide a footnote.**

We thank the reviewer for his/her thorough and detail review, we have checked tables and figures and now all numbers do match.

**S2 Table.
Beverages T0 n is 686 whereas Table S2 states 688**

Fixed.

**Flavoured is spelled both with and without a "u" suggest the American
English spelling without a "u" to be consistent.**

Done.

**Milks & milk-based drinks - too many uses of the word "powder".**

Extra powders have been removed.

**Yogurts - no n after Longitudinal.**

Done.

**Breakfast cereals - flakes not lakes.**

Done.

**Desserts & ice cream - vegetable not vegetables and chips not chip.**

Done.

**Candies & sweet confectioneries - change carcass for coating.**

Done**.**

**Sweet spreads - Unclear on the difference between "honey" and honey (all kinds).**

We have removed “honey”.

**Savory baked products - suggest starting all sections with a capital
letter but if not Christmas should have one. Is Christmas bread
savory?**

Christmas bread is a sweet baked product and is now listed in the right place. All sections start with a capital letter right now.

**Savory spreads - should hicken be chicken? Unclear why broth is in the
spread category not soups.**

Change made. In Chile, broths are more typically used for seasoning food preparation (i.e. for preparing rice or chicken stew) rather than as a soup; therefore, they were included in the savory spread’ group.

**Ready to eat meals - Longitudinal should be on new line**

Done.

**S3 Table
There is a semi-colon in the first list (after ice creams) that makes
it unclear which groups you are referring to. Perhaps a value is
missing?**

A value was indeed missing, we have added it in the current version.

**Also a full stop after sausages that should not be there.
Oxford comma after confectionery in iv. No space after > before 8000mg**

Done.
